# ADVANCING HEARING HEALTH EQUITY FOR OLDER ADULTS: FINDINGS FROM THE HEARS RCT

**DOI:** 10.1093/geroni/igac059.948

**Published:** 2022-12-20

**Authors:** Carrie Nieman, Joshua Betz, Emmanuel Garcia Morales, Jonathan Suen, Nicole Marrone, Hae-Ra Han, Sarah Szanton, Frank Lin

**Affiliations:** Johns Hopkins University School of Medicine, Baltimore, Maryland, United States; Cochlear Center for Hearing & Public Health, Baltimore, Maryland, United States; Cochlear Center for Hearing and Public Health, Baltimore, Maryland, United States; Johns Hopkins University, Baltimore, Maryland, United States; University of Arizona Department of Speech, Language, and Hearing Sciences, Tucson, Arizona, United States; Johns Hopkins University, Baltimore, Maryland, United States; Johns Hopkins University School of Nursing, Baltimore, Maryland, United States; Johns Hopkins Cochlear Center for Hearing and Public Health, Baltimore, Maryland, United States

## Abstract

Hearing loss is highly prevalent but disparities exist in hearing care. Delivering care in partnership with community health workers (CHWs) is an established approach to addressing disparities but has not been robustly studied in hearing care. We recruited older adults with hearing loss from community sites in Baltimore, MD. The 2-hour intervention consists of fitting a low-cost amplification device and counseling.151 participants were randomized. The primary outcome was change in communication function (Hearing Handicap Inventory for the Elderly-Screening [HHIE-S]; range 0-40; higher scores indicate poorer function). Communication function significantly improved in the intervention group, with an intention to treat estimated average treatment effect of a -12.98 point change (95% CI: -15.52, -10.42). In the first randomized control trial of a CHW-delivered hearing care intervention for older adults using low-cost amplification devices, participants receiving the intervention demonstrated a treatment effect comparable to prior studies of conventional hearing aids fit by audiologists.

